# Nutritional properties of raw and cooked *Azolla caroliniana* Willd., an aquatic wild edible plant

**DOI:** 10.1002/fsn3.3904

**Published:** 2024-01-10

**Authors:** Daniel Winstead, Francesco Di Gioia, Marjorie Jauregui, Michael Jacobson

**Affiliations:** ^1^ Department of Ecosystem Science and Management, College of Agricultural Sciences The Pennsylvania State University University Park Pennsylvania USA; ^2^ Department of Plant Science, College of Agricultural Sciences The Pennsylvania State University University Park Pennsylvania USA; ^3^ Department of Food Science, College of Agricultural Sciences The Pennsylvania State University University Park Pennsylvania USA

**Keywords:** boiling, natural fermentation, pressure cooking, protein digestibility, total phenolic content, wild edible plants

## Abstract

*Azolla caroliniana* Willd. is an understudied wild edible plant native to the Eastern United States. Other species of *Azolla* have been used across the world for several thousand years as a livestock feed and as “green manure.” The use of *Azolla* for human consumption is thought to be limited by its high total polyphenolic content (TPC). However, the TPC and nutritional content of *A. caroliniana* has not been thoroughly studied. We measured TPC and other nutrients before and after cooking methods designed to lower TPC. We found that TPC was 4.26 g gallic acid equivalent (GAE) kg^−1^ DW in raw *A. caroliniana*. All cooking methods significantly lowered TPC. Protein content was 19% DW, and the apparent protein digestibility was 78.45%. Our yield was 173 g FW m^−2^ day^−1^ and 5.53 g DW m^−2^ day^−1^. *Azolla caroliniana* is a high‐yielding plant with great potential for cultivation and domestication.

## Graphical abstract

*Azolla caroliniana* is an underutilized wild edible plant with many uses. After testing various postharvest treatments, we show that this species native to North America is nutritious and not limited by high antinutritional factors like its congeners.

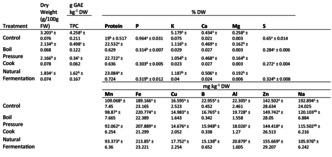

## 1 INTRODUCTION

*Azolla* Lam., also called water fern, mosquito fern, or “azolla,” is a genus of floating fern which has been cultivated for several thousand years (particularly across Asia) comprised six described species native to most tropical and temperate biomes across the world (Small, 2013). *Azolla* has received increasing attention in the last few decades because of its many potential uses as bioremediation, wastewater treatment, “green” manure, biodiesel production, livestock feed, mosquito control, and human food (Brouwer et al., 2018; Carlozzi & Padovani, 2016; Katayama et al., 2008; Miranda et al., 2016; Pouil et al., 2020; Small, 2013). The incredibly diverse set of uses for *Azolla* prompts further attention and research. The high‐protein content and fast‐growing capability of *Azolla* makes it a great option for quick food production. *Azolla* grows and reproduces very quickly, allowing some species (*Azolla filiculoides* and *A. pinnata*) to produce as much as 100 mg DW ha^−1^ per year in natural habitats (Miranda et al., 2016). Even the use of *Azolla* as “green manure” is not new to human history as it has been used to fertilize grain crops for hundreds of years and is currently produced in large tarp‐bottomed ponds and used to supplement diets for pigs, ducks, rabbits, fish, cattle, and chickens with positive effects on their nutrition (Wagner, 1997).

Although similar in appearance, physical properties, and opportunities for use to the more commonly known flowering plant, duckweed (members of the subfamily Lemnoideae), *Azolla* is a true fern (Polypodiopsida) and has several distinct characteristics that set it apart from duckweed. First, *Azolla* has an obligate mutualism with nitrogen fixing cyanobacteria giving it the ability to grow in more nutrient deficient areas than duckweed and even to replenish nutrient loads of agricultural systems (Yao et al., 2018). Importantly, *Azolla* can fix nitrogen twice as fast as *Rhizobia* spp. in soybeans (Pouil et al., 2020). Its ability to efficiently fix nitrogen is itself enough of a reason to justify *Azolla*'s study. Additionally, studies suggest *Azolla* may be more cool and shade tolerant than duckweed (Muradov et al., 2014). *Azolla* has also been shown to have a higher relative growth rate to some species of duckweed (Hamdan & Houri, 2022). These differences between duckweed and *Azolla* justify further investigation of *Azolla* separately and in tandem with duckweed.

Like other wild edible plants native to the United States, characteristics of *Azolla* species make them good candidates as a nutrition security resource and for food resilience options (Winstead et al., 2023). Some research has already been done on *Azolla*'s potential use in the space program because of its unique attributes and low space requirements (Katayama et al., 2008). However, given *Azolla*'s fast growth rate, nutritional profile, and its ability to be grown in both indoor and outdoor settings suggest that it is a good candidate for use during times of food insecurity more broadly. Whether it be for a “quick fix” solution in catastrophe scenarios or long‐term resilience plan, *Azolla* has the potential to provide large amounts of protein and calories for people and livestock. If systems for *Azolla* cultivation and preparation can be made more efficient, its indoor or outdoor cultivation after natural disasters could provide climate resilient supplemental nutrient production.

Unfortunately, the use of *Azolla* as feedstock and human food is thought to be limited by its high polyphenolic content (Brouwer et al., 2018; Everette et al., 2010). Polyphenols at lower concentrations are beneficial because of their antioxidant activity, however, high concentrations of polyphenols can limit nutrient absorption in the body and act as antinutritional factors (Hassan et al., 2020; Mennen et al., 2005). More specifically, polyphenols bind and precipitate proteins and carbohydrates, making them biologically inaccessible (Bravo, 1998). The measured total phenolic content (TPC) of *Azolla* species of 23.8–78.7 g GAE kg^−1^ DW is 5–10 times the amount in most other fruits and vegetables (Bravo, 1998; Brouwer et al., 2018). Although antioxidant activity can come from several metabolites, evidence suggests that antioxidant activity in *Azolla* species is mostly dependent on phenolic compounds allowing TPC to be a good proxy for antioxidant activity (Hassan et al., 2020).

There are several cooking methods that have been shown to decrease polyphenol content including, but not limited to, boiling, pressure cooking, fermenting, and sautéing (Samtiya et al., 2020). These cooking methods also increase the digestibility of the polyphenols themselves in addition to allowing for the continued bioavailability of other macronutrients (Bravo, 1998). It is unknown how these cooking methods affect the nutrition of *Azolla* species and whether they would reduce polyphenol content to a level that would reduce its ingestion limitations. These methods are simple and low cost, and could be a simple solution for enabling the use of *Azolla* as a food source in many situations.

However, net losses in the nutritional quality of raw vegetables usually occur after cooking processes are used to convert nutrients from being inaccessible to digestible. Multiple studies have pointed out that traditional cooking methods, such as boiling, and microwaving can affect the phenolic content and antioxidant capacity of popular vegetables such as kale, broccoli, and cabbage (Abushita et al., 2000; Sahlin et al., 2004; Stewart et al., 2000; van Het Hof et al., 2000; Zhang & Hamauzu, 2004). However, research conclusions about whether these cooking methods increase or decrease the phytochemical properties of the vegetables are not consistent. The effects of cooking methods on the content and bioavailability of nutrients in vegetables are variable and depend not only on the type of vegetable, but also on the complexity of the food matrix and the cooking method involved (Boari et al., 2013; Turkmen et al., 2005; Ur‐Rehman et al., 2003). Depending on the characteristics of the cooking methods, these can cause several changes in the physical and chemical properties of the foods (Turkmen et al., 2005; Ur‐Rehman et al., 2003).

The solubility of polyphenol groups in water is determined by their polar properties (Haminiuk et al., 2014). If food matrices containing water‐soluble compounds are subjected to water‐based cooking methods, their phenolic content is more likely to decrease, due to a leaching effect (Price et al., 1997). Cooking methods such as boiling, frying, and pressure cooking involve temperatures that can go up to 190°C. At these high temperatures key nutrients may be reduced; however, their cooking time is often shorter, which is better for the retention of nutrients compared to slower methods. Some studies have also found that during heat treatments the formation of antioxidant phenolic compounds may occur (Giovanelli et al., 2002; Polat et al., 2022).

Two commonly studied species, *A. filiculoides* Lam. and *A. pinnata* R.Br., are invasive and considered noxious weeds in their non‐native habitats (Small, 2013). Between these two species, *A. filiculoides* has a significantly lower concentration of polyphenols than *A. pinnata* (Brouwer et al., 2018). Since the literature suggests that there may be a significant difference in phenolic content between species, it would be worthwhile to investigate other species in the *Azolla* genus to determine their phenolic content. Additionally, most *Azolla* nutritional research focuses on *A. pinnata* and *A. filiculoides*, leaving more to be discovered about other *Azolla* species such as the native species of *Azolla* to the eastern United States, *A. caroliniana* Willd. (commonly known as Carolina azolla). Although *A. caroliniana* has been argued to be conspecific with *Azolla cristata* Kaulf. (Evrard & Van Hove, 2004), lack of definitive evidence and the predominant use of the name *A. caroliniana* in recent literature warrants our continued use of the name *A. caroliniana* until further investigation (Bunch & Hayden, 2020).

To investigate this lesser known *Azolla* species and to decrease the risk of spreading non‐native plants in the Pennsylvania study area, we used *A. caroliniana* for this study. We tested three cooking methods (boiling, pressure cooking, and natural fermentation) that have been shown to decrease polyphenolic content in foods by multiple studies, with the aim of reducing antinutritional factors potentially restricting consumption of *Azolla* by both humans and livestock (McLachlan & Landman, 2013; Samtiya et al., 2020, 2021). These treatments were chosen because of their simple, low‐cost nature and their potential for decreasing the TPC of *Azolla* and increasing its nutritional value. Other nutritional values were also measured and analyzed after postharvest treatments and compared using nonparametric statistical tests.

## 2 METHODOLOGY

### 2.1 Plant material and growing conditions

*Azolla caroliniana* was obtained from PondPlantsOnline.com and was rinsed with tap water and inspected for stray duckweed and other contaminants upon delivery. The *A. caroliniana* was then acclimated for 4 days in the greenhouse without supplemental lighting. The growth conditions for this experiment as well as the harvest technique and nutrient solution were augmented from the Brouwer et al. (2018) study protocol using *A. filiculoides* and *A. pinnata*. The *A. caroliniana* was grown under controlled environmental conditions in a greenhouse located at Penn State's University Park Campus in State College, Pennsylvania. Supplemental lighting was provided by VYPR 3p Broad Indoor LED lights (Fluence, Austin, TX) dimmed to 30% capacity. This increased photosynthetic photon flux density (PPFD) by 230 μmol m^−2^ s^−1^ when hung 115 cm from water surface. Supplemental lights were turned on daily for 16 h starting at 6 a.m. The daytime temperature range was set between 23 and 27°C and night temperature range between 20 and 24°C.

The *A. caroliniana* was grown within four aerated 60‐L opaque plastic containers, each with a net surface area of 4505 cm^2^ and depth of 14 cm filled with nutrient solution specified below. Plastic foam‐covered wire was used to decrease surface agitation of the water around the aeration system. The *A. caroliniana* was grown for 14 days until the complete surface area of the water was covered by *Azolla*. The slightly augmented nutrient solution from Brouwer et al. (2018) was created with deionized (DI) water and macronutrients: 0.7 mM KNO_3_, 0.1 mM Ca(NO_3_)_2_.4H_2_O, 0.13 mM KH_2_PO_4_, and 0.1 mM MgSO_4_.7H_2_O; and micronutrients: 4.7 μM FeNa‐EDTA, 2.2 μM MnSO_4_.H_2_O, 0.1 μM Na_2_Mo_4_.2H_2_O, 8.1 μM H_3_BO_3_, 0.06 μM CuSO_4_.5H_2_O, and 3.1 μM ZnSO_4_.H_2_O. The complete 60 L of solution in each container was replaced after the first harvest on July 14, 2022. Nutrient solution levels were replenished daily in each container from water loss due to evaporation with DI water to maintain water level and depth.

### 2.2 Harvest and postharvest processing treatments

The *A. caroliniana* was harvested four times over a span of 16 days from each of the four 60‐L basins. For each harvest day, four 100 g samples were harvested using a mesh strainer from each basin and were treated with one of the four treatments. Treatments are fully described in Table 1. The four basins served as biological replicates and each harvest served as a repeated experiment. This resulted in a total sample size of *n* = 64, with 16 samples for each treatment.

**TABLE 1:** Postharvest treatments.

Treatment name and abbreviation	Sample FW mass (g)	Treatment description
Control (C)	100	Rinsed with DI water to remove nutrient solution residue and stored in sealable freezer bag.
Boiling (B)	100	Rinsed with DI water to remove nutrient solution residues. Sample placed in 1 L of distilled water in an Instant Pot brand 6‐qt pressure cooker and set to “Sauté” for 20 min to boil uncovered. *Azolla* was strained with mesh strainer and water discarded. Boiled material was cooled for 5 min at room temperature and transferred to sealable freezer bag and remaining water gently squeezed out.
Pressure cooking (P)	100	Rinsed with DI water to remove nutrient solution. Place sample and 1 L of distilled water into an Instant Pot brand 6‐qt pressure cooker and pressure cooked on “high” for 1 min. Steam released using “Quick Release” method. *Azolla* was strained with mesh strainer and water discarded. Pressure cooked material was cooled for 5 min at room temperature and transfer to sealable freezer bag and excess liquid gently squeezed out.
Natural fermentation (F)	100	Rinsed with DI water to remove nutrient solution. Room temperature distilled water and each 100 g *Azolla* sample were added to a small blender to equal 400 ml total (enough to cover the *Azolla*). The water and sample are then blended for 3 sec with a blender on high and placed into 1 L glass jars. Plastic bags filled with distilled water were added on top of the *Azolla* in the jars to ensure that the blended *Azolla* stayed submerged. The jars were then sealed and placed in room temperature for 48 h to naturally ferment. After 48 h, the *Azolla* was strained with cheese cloth, excess liquid gently squeezed out, and transferred to sealable freezer bag.

### 2.3 Freeze‐drying and storage

Treated samples were prepared for nutrient analysis by freezing to −80°C, then freeze‐drying. Dry samples were weighed and then ground in a bladed, spice grinder until homogenized (about 6 s on the highest setting). Ground samples were labeled and placed into resealable mylar bags and stored in a −20°C freezer until analyzed.

### 2.4 Nutrient analysis

The crude protein content (CP) of freeze‐dried and ground *Azolla* samples was calculated by multiplying the total nitrogen content with the conversion factor of 4.9, which has previously been measured for *Azolla* species (Brouwer et al., 2018). The moisture levels of the dried samples were determined by using an infrared moisture analyzer (Sartorious MA37, Germany).

The TPC of the *Azolla* was measured using the Folin–Ciocalteu method with two technical replicates for each sample (Waterman & Mole, 1994). Crude lipid contents of other species of *Azolla* are roughly about 100 g kg^−1^ DW (Brouwer et al., 2018). To more accurately measure TPC, lipids were removed from 500 mg of freeze‐dried and ground samples by extraction with hexane for 15 min stirred at room temperature. Hexane was then removed by filtering the samples using a Büchner funnel with no. 1 Whatman paper. This step was repeated once more. For sample extractions, dried residues were suspended in 10 mL of acetone:water:acetic acid (80:20:0.1, v/v/v) and stirred for 15 min at room temperature. The extraction step was repeated three times total. All filtrates were combined and evaporated to near dryness in a centriVap concentrator (Labconco, USA). Volume of final filtrates were measured and 2 mL of ultrapure water was added to each sample to aid in vortexing samples to collect any possible remaining polyphenols on walls of drying tubes. Samples were then spun in a centrifuge to pellet particulates and remaining liquid was filtered twice through 40 μm nylon filters. An aliquot of 40 μL was diluted with 1560 μL of ultrapure water and added to 100 μL of Folin–Ciocalteu reagent. After 5 min in the dark, 300 μL (200 g L^−1^) of Na_2_CO_3_ solution was added. The solution was mixed and incubated in a water bath for 30 min at 37°C. Then, after mixing and cooling at room temperature, absorbance was measured at 765 nm using a microplate spectrophotometer (Multiskan GO microplate Spectrophotometer, Thermo Fisher Scientific, Vantaa, Finland). Gallic acid was used as the calibration standard, and TPC is expressed in gallic acid equivalence.

The freeze‐dried samples were then passed through a size 35 mesh sieve and sent to the Agricultural Analytical Services Laboratory at University Park, PA, for mineral and nitrogen analyses. Samples were analyzed for total nitrogen using dry combustion with an Elementar Max Cube in CN mode (Elementar Americas Inc., Ronkonkoma, NY) as described in Vecchia et al. (2020). Additionally, the macrominerals (P, K, Ca, Mg, S, and Na) and microminerals (Mn, Fe, Cu, B, and Zn) were measured after acid digestion (Huang & Schulte, 1985) using an ICP‐OES (Varian 730‐ES, Agilent Technologies, Santa Clara, CA, USA). Nutrient and mineral profiles were expressed on a dry weight (g or mg kg^−1^) and fresh weight basis (g or mg 100 g^−1^ FW *Azolla*).

Percent apparent protein digestibility (APD%) was calculated using the assay created by Hsu et al. (1977). We prepared an enzyme solution by dissolving 16 mg of Trypsin (type IX‐S) from porcine pancreas (13,000–20,000 BAEE units mg^−1^ protein), 31 mg of α‐chymotrypsin type II from bovine pancreas (≥40 units mg^−1^ protein), and 13 mg of pepsin from porcine gastric mucosa (≥3200 units mg^−1^ protein) in 5 mL distilled water at 37°C for 10 min. The pH was adjusted to 8 using 0.1 M HCl or 0.1 M NaOH. Then we brought the final volume of the enzyme solution to 10 mL, and pH was adjusted down to 8 again. We measured out each sample so that it contained 10 mg of nitrogen and dissolved them in 7 mL of distilled water in duplicate. Then we adjusted the pH to 8 using the same method used for the enzyme solution. Samples were then soaked for 60 min at 37°C in water bath. After the water bath step, 1 mL of enzyme mix was added to samples, vortexed, and incubated for 10 min at 37°C. We measured the pH exactly 10 min after placing in water bath. Bovine serum albumin (BSA) was used as a positive control for APD%. The apparent protein digestibility was then calculated using the following equation where *X* = pH after 10 min:

Percent apparent protein digestibility=210.46−18.10×X.

### 2.5 Statistics and data analysis

A Shapiro–Wilk test was run for all dependent variables for each treatment to test for normality. Many of the groups were nonparametric, so nonparametric tests were used for all tests to decrease sensitivity and ensure all assumptions were met. Kruskal–Wallis tests (nonparametric ANOVA) were performed for each of the dependent variables against the four‐level treatment independent variable. If Kruskal–Wallis tests showed a significant difference, post hoc pairwise Wilcoxon tests (nonparametric *T* tests) were performed to determine pairwise differences among treatments. All *p* values were adjusted using Bonferroni's correction. The alpha level was set to *α* = .05.

To measure the sampling adequacy before conducting the principal component analysis (PCA), Kaiser–Meyer–Olkin (KMO) and Bartlett's sphericity test were performed using the “KMO” and “BARTLETT” commands in R using the EFAtools package. The KMO value was 0.709, and the Bartlett's sphericity test was significant (*p* < .001; χ^2^ = 406) suggesting that the dataset met the criteria for factor analysis and for using PCA as a data reduction technique. The PCA was performed in R using command “prcomp” to summarize variance observed within the dataset.

## 3 RESULTS

Over the 30‐day grow‐out period of this experiment, we were able to achieve an *Azolla* yield of 173 g FW m^−2^ day^−1^ and 5.53 g DW m^−2^ day^−1^. If extrapolated to larger surface areas, this is equivalent to 20.16 Mg ha^−1^ year^−1^ DW. Fresh *A. caroliniana* samples had a mean water content of 96.8%. A Kruskal–Wallis test on dry weight shows that treatment type did have a significant effect on final dry weight. Post hoc pairwise Wilcoxon tests revealed that all treatment methods were significantly lower in final dry weight than the control, where fermentation had a 42.74% decrease in dry weight mass, and boiling and pressure cooking had a 32.88% decrease in dry weight mass on average. This loss of material is expected for any treatment, especially those involving submersion in water.

### 3.1 TPC content and yield

TPC of raw *A. caroliniana* was 4.26 g GAE kg^−1^ DW. Kruskal–Wallis tests revealed a significant difference in TPC between the postharvest treatments (*p* < .001). Post hoc pairwise Wilcoxon tests showed that there were significant differences between the control and all other treatments, and that boiling and pressure cooking both had significantly lower TPC than natural fermentation. TPC was reduced by about 88.3%, 92%, and 62% with boiling, pressure cooking, and natural fermentation, respectively, compared to the control. Likewise, total dry weight content and yield of polyphenolics significantly decreased with all cooking methods (Tables 2 and 3).

**TABLE 2:** Effect of postharvest treatments on nutrients by dry weight.

Treatment	Dry weight (g 100 g^−1^ FW)	g GAE kg^−1^ DW	% DW					
		TPC	Protein	P	K	Ca	Mg	S
Control	3.203^a^ ± 0.076	4.258^a^ ± 0.211	19^b^ ± 0.517	0.964^a^ ± 0.031	5.179^a^ ± 0.075	0.434^a^ ± 0.021	0.258^a^ ± 0.003	0.65^a^ ± 0.014
Boil	2.134^b^ ± 0.068	0.498^c^ ± 0.122	22.532^a^ ± 0.629	0.314^b^ ± 0.007	1.116^b^ ± 0.029	0.469^a^ ± 0.027	0.162^b^ ± 0.003	0.284^c^ ± 0.006
Pressure cook	2.166^b^ ± 0.078	0.34^c^ ± 0.062	22.722^a^ ± 0.636	0.303^b^ ± 0.005	1.054^b^ ± 0.023	0.468^a^ ± 0.027	0.164^b^ ± 0.003	0.272^c^ ± 0.004
Natural fermentation	1.834^c^ ± 0.074	1.62^b^ ± 0.167	23.084^a^ ± 0.724	0.319^b^ ± 0.012	1.187^b^ ± 0.04	0.506^a^ ± 0.024	0.192^b^ ± 0.006	0.324^b^ ± 0.008

	mg kg^−1^ DW						
	Mn	Fe	Cu	B	Al	Zn	Na
Control	109.068^a^ ± 7.45	189.166^a^ ± 23.165	16.595^a^ ± 2.523	22.955^a^ ± 0.452	22.305^a^ ± 2.461	142.502^a^ ± 28.634	192.894^a^ ± 24.025
Boil	98.87^a^ ± 7.665	220.774^a^ ± 22.389	14.983^a^ ± 1.643	16.765^b^ ± 0.342	19.728^a^ ± 1.558	149.742^a^ ± 28.05	120.103^ab^ ± 6.884
Pressure cook	92.062^a^ ± 6.254	207.889^a^ ± 21.299	14.676^a^ ± 2.052	15.948^b^ ± 0.338	18.026^a^ ± 1.27	144.418^a^ ± 26.513	115.502^ab^ ± 6.216
Natural fermentation	93.373^a^ ± 6.36	213.85^a^ ± 23.221	17.752^a^ ± 2.254	15.138^b^ ± 0.652	20.879^a^ ± 1.605	155.669^a^ ± 29.207	105.976^b^ ± 6.242

*Note*: Values are mean ± SE (*n* = 16). Superscript letters are significance letters, used to denote significant differences between treatments.

**TABLE 3:** Average yield from each 100 g sample of fresh *Azolla* after postharvest treatment (mg).

Treatment	TPC (GAE)	Protein	P	K	Ca	Mg	S	Mn	Fe	Cu	B	Al	Zn	Na
Control	13.57^a^ ± 0.7	60.86^a^ ± 2.22	3.09^a^ ± 0.13	16.62^a^ ± 0.53	1.39^a^ ± 0.07	0.83^a^ ± 0.02	2.08^a^ ± 0.07	350.81^a^ ± 26.62	602.95^a^ ± 71.92	53.39^a^ ± 8.68	73.7^a^ ± 2.66	71.18^a^ ± 7.6	463.63^a^ ± 102.15	626.99^a^ ± 86.64
Boil	1.13^b^ ± 0.32	47.87^b^ ± 1.7	0.67^b^ ± 0.03	2.4^b^ ± 0.13	1.01^b^ ± 0.08	0.34^b^ ± 0.01	0.61^b^ ± 0.02	213.74^b^ ± 19.61	470.92^ab^ ± 48.88	31.14^b^ ± 2.99	35.9^b^ ± 1.59	42.12^b^ ± 3.8	304.13^a^ ± 46.66	255.74^b^ ± 16.94
Pressure cook	0.73^b^ ± 0.14	49.42^b^ ± 2.57	0.66^b^ ± 0.03	2.3^b^ ± 0.11	1.01^b^ ± 0.06	0.35^b^ ± 0.01	0.59^b^ ± 0.02	197.63^b^ ± 13.61	445.42^ab^ ± 44.39	32.21^ab^ ± 4.73	34.53^b^ ± 1.42	39.07^b^ ± 3.16	317.11^a^ ± 57.3	250.21^b^ ± 17.25
Natural fermentation	2.91^b^ ± 0.26	42.78^b^ ± 2.65	0.59^b^ ± 0.04	2.2^b^ ± 0.15	0.92^b^ ± 0.05	0.35^b^ ± 0.01	0.6^b^ ± 0.04	172.28^b^ ± 14.23	389.42^b^ ± 42.43	33.87^ab^ ± 5.6	27.4c ± 1.11	39.36^b^ ± 4.32	307.5^a^ ± 72.18	195.26^b^ ± 16.13

*Note*: Values are mean ± SE (*n* = 16). Superscript letters are significance letters, used to denote significant differences between treatments.

### 3.2 Protein content and yield

The total nitrogen content of raw *Azolla* samples was on average 3.88% ± 0.1%. The average protein content of raw *A. caroliniana* of 19.0% ± 0.5% was similar to that of both *A. filiculoides* and *A. pinnata*, which have been estimated at 19.5% ± 1.8% and 17.6% ± 1.6%, respectively (Brouwer et al., 2018). The total protein contents for postharvest treatments were statistically different showing that all postharvest treatments increased in percent protein on a dry weight basis. However, when comparing protein yield per 100 g of fresh *A. caroliniana*, there is a loss of protein from postharvest treatments by 23.3% on average from all treatments compared to the control. There was no significant difference in protein content between the three noncontrol treatments.

The APD% calculated for the positive control (BSA) was 89.24%. Unfortunately, due to an unexpected sample loss, APD% could only be calculated for raw *A. caroliniana* and not for other treatments. The APD% of raw *A. caroliniana* was 78.45% ± 1.63%, *n* = 16. This equates to 14.91 g of digestible protein 100 g^−1^ DW raw *A. caroliniana*.

### 3.3 Mineral content and yield

Raw *Azolla caroliniana* contains moderate levels of sodium compared to other common vegetables but is lacking in other minerals when in relation to raw fresh weight (Rickman et al., 2007). Most DW mineral levels decreased following postharvest treatments aside from some having no significant difference (Ca, Mn, Fe, Cu, Al, Zn). All postharvest treatments showed a significant loss in mineral yield from the control except for Zn, which showed no significant change. Boiled and pressure‐cooked samples were statistically identical for all mineral contents and yields. Additionally, mineral content was not statistically different between the three cooking methods except for S content, which did not decrease as much in fermentation as with boiling and pressure cooking. Likewise, mineral yields from cooking methods were not statistically different from each other except for B, in which case fermentation reduced yield significantly more than boiling or pressure cooking.

### 3.4 Principal component analysis

**FIGURE 1:**
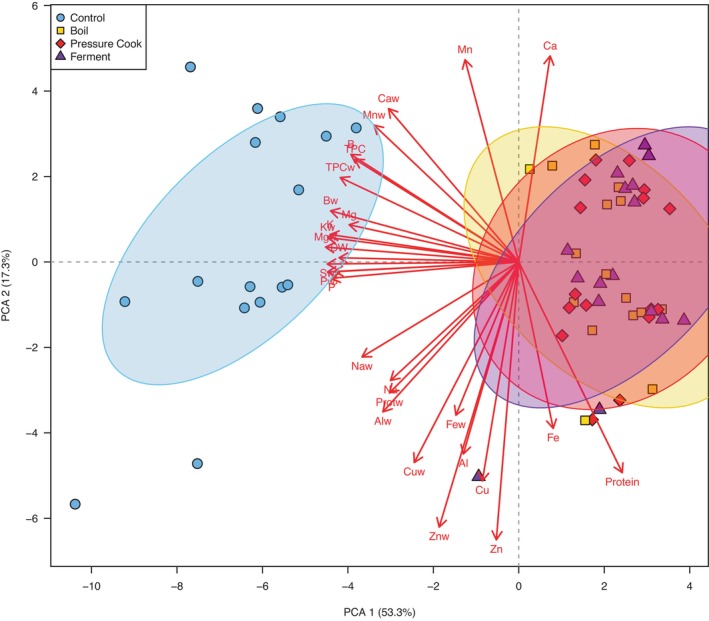
PCA biplot (PC1 vs. PC2) showing spatial distribution of all dependent variables and four treatment levels. “w” denotes yield per 100 g fresh weight. All others are dry weight content. DW, dry weight; Prot, protein; TPC, total phenolic content. Ellipses show 95% confidence for variable of respective color.

The PCA was conducted using all data from all treatments and color coded to better visualize variance between treatments described previously (Figure 1). PC1 explained 53.5% of the data's variance, while PC2 accounted for 17.3% of the data's variance. The control was associated with greater dry weight content and wet weight yield for TPC, dry weight, and most minerals. The three cooking methods are largely indistinguishable, but are all associated with higher dry weight contents of iron, protein, and calcium. Overall, the PCA shows clear separation of the control from all the treatments tested; however, little distinction is observed between cooking methods, suggesting that all three cooking methods had similar effects on the nutritional value of *A. caroliniana*.

## 4 DISCUSSION

### 4.1 Raw *Azolla caroliniana* nutrition

Our results suggest that the TPC of *A. caroliniana* was lower compared to other species of the genus *Azolla*, this increases the usability of *A. caroliniana* for all applications as its raw TPC is comparable to many other raw fruits and vegetables, meaning the intake of this plant will likely not be limited by TPC as an antinutritional factor as for other species of *Azolla* (Álvarez et al., 2016).

Our results also corroborate with similar studies showing similar protein contents of *Azolla* species (Brouwer et al., 2018; Kaur et al., 2015). The moderate protein content of *A. caroliniana* suggests that it can be used as a source of protein for those with protein poor diets, and as a protein feed for livestock given its full amino acid profile (Brouwer et al., 2018). Although the water content of *A. caroliniana* is quite high (96.8%), it is incredibly fast growth rate still allows its dry mass yield to be close to, or exceed, dry mass production yields of most global commercially grown crops, like potatoes, maize, and soybeans (Ritchie et al., 2022). Concurrently, our estimated growth rate of 20.16 Mg ha^−1^ year^−1^ is similar to other studies investigating dry mass yields of other *Azolla* species (Debusk & Reddy, 1987; Miranda et al., 2016; Muradov et al., 2014; Vincenzini et al., 1985). Our small‐scale, pilot experiment shows how easy growing *Azolla* species could be for any community/family scale operation.

The protein content of raw *Azolla* is on average 19%, and though this is lower than that of microgreens such as peas (46.9%) (Poudel et al., 2023), *A. caroliniana* does not require new seed material after harvest as it is propagated vegetatively. This advantage means that the yield of protein from *Azolla* may be higher and more accessible, especially in resource‐limited environments or during disasters. Although its protein content is not much higher than the average vegetable by dry weight, its production yield per area over time is high and would require less fertilizer given its nitrogen‐fixing ability (Fujihara et al., 2001).

Apparent protein digestibility revealed that the protein of raw *Azolla* is moderately digestible compared to common grain proteins. It is known that the presence of tannins and similar molecules decrease protein digestibility (Brouwer et al., 2018; Mennen et al., 2005). Therefore, although we were unable to perform the protein digestibility assay on noncontrol treatments, we presume that APD% would be higher in cooked samples due to decreased TPC. Future experiments should prioritize measuring the effect of cooking treatments on APD%.

The TPC of raw *A. caroliniana* falls within the range of other common vegetables and was much lower than in other species of *Azolla*, and therefore *A. caroliniana* is likely much more usable and less limited by antinutritional factors (Álvarez et al., 2016; Brouwer et al., 2018). Because of this significantly lower TPC which fall in the range of many other fruits and vegetables, the TPC of *A. caroliniana* could be considered a beneficial and marketable attribute as being high in antioxidants (Dryden et al., 2006). When looking at mineral content of raw *Azolla* on a dry weight basis, it has higher mineral contents than many other vegetables (Table 4) (Marles, 2017). Notably, it is high in potassium and phosphorus on a dry mass basis.

**TABLE 4:** Mineral content comparison to other common vegetables by dry weight.

Vegetable	Ca	K	Mg	P	Cu	Fe	Mn	Zn
*Azolla caroliniana*	4.3	51.8	2.6	9.6	0.017	0.19	0.11	0.14
Wheat	0.8	7.3	2.2	9.1	0.014	0.16	0.09	0.10
Rice, brown	0.6	3.2	1.7	5.0	0.007	0.06	0.04	0.03
Maize, sweet	0.7	15.6	2.8	6.3	0.003	0.03	NA	0.06
Barley	0.7	5.9	1.5	42.0	NA	NA	NA	NA
Common bean	4.3	24.9	3.3	8.4	0.014	0.12	0.03	0.06
Soybean	3.2	23.2	3.1	9.4	0.020	0.20	0.06	0.07
Sweet potato, raw	1.5	14.5	0.9	1.8	0.007	0.06	0.03	0.01
Broccoli	5.1	NA	3.7	NA	NA	NA	NA	NA
Tomato, ripe raw	1.8	48.3	2.1	5	0.001	0.08	0.02	0.03
Papaya, ripe	2.9	23.1	2.3	1.5	0.008	0.15	0.00	0.03

*Note*: Values expressed in g kg^−1^ DW. Shadow is to highlight *Azolla*, the study organism, apart from the other foods.

Raw *A. caroliniana* contains 14.91 g digestible protein 100 g^−1^ DW. Our measurement suggests that 335 g of dried, raw *Azolla* is enough to provide an adult person's daily protein requirement of 50 g a day (Liu et al., 2017). Given a yield of 5.53 g DW m^−2^ day^−1^, and considering the recommended protein daily intake, 1 ha of *Azolla* can provide enough digestible protein to fulfill the daily protein requirement for 165 people every year.

### 4.2 Effect of treatments

Our findings show that all three low‐cost treatment methods can improve the nutritional quality and accessibility of *Azolla* species by lowering TPC. Boiling, pressure cooking, and natural fermentation are all viable treatment methods that can lower TPC of high‐TPC *Azolla* species. However, boiling and pressure cooking were more effective at lowering TPC than natural fermentation. Energy requirements for boiling and pressure cooking are high and may not be suitable in low resource conditions, in which case natural formation may be the best low‐cost, low‐energy solution to decreasing TPC in *Azolla* species. As we have shown, there is a large decrease in TPC from all treatments in relation to the control, and though there was a significant decrease in total protein for all treatments, total protein yield only dropped an average of 23.28% on a fresh weight basis for all treatments compared to fresh *A. caroliniana*.

Because of the lower TPC of *A. caroliniana*, the results of the treatments are not as directly relevant as once thought. However, these cooking methods and data may be used to inform others on the processing of the other more polyphenol‐rich species such as *A. filiculoides* and *A. pinnata*.

As indicated by the PCA and statistical tests, our results show that there are very few differences between mineral contents and yields between these cooking methods. Additionally, there does seem to be a consistent loss of nutrients using water submersion‐based cooking methods, however this is expected as the goal of this experiment was in reducing TPC while retaining most of the protein. Previous studies show how fermenting *Azolla* breaks down fiber and antinutritional factors while retaining levels of zinc, copper, and chromium; increasing its usability through lactic acid fermentation (Cruz et al., 2011). Our study corroborates this finding. Fermentation also retains protein and amino acids, and when fed to chickens resulted in a significant increase in the perceived palatability of the chicken meat (Nuraini et al., 2022). Fermented *Azolla* livestock feeds have also been shown to improve growth rate of tilapia fed with feed mixes with 20% fermented *A. caroliniana* (Hundare et al., 2018).

The taste profile of raw and cooked *Azolla* for human consumption has been described by chefs in the past as being “crisp and juicy, without much flavor but tasting somewhat of earth, metal, minerals, mushrooms, moss, and grass,” and dried *Azolla* as being “reminiscent of green tea, buttercup, and kelp” (Sjödin, 2012). Although this study did not take into consideration the sensorial properties of the *A. caroliniana*, we noted aromas like that of moss, earth, and seaweed during harvesting and cooking of *A. caroliniana*. More notably, after the natural fermentation treatment, we noticed the *A. caroliniana* had a sweet and aromatic scent which remained even after freeze‐drying and was considered appetizing to some. This cooking method has not been mentioned in previous studies and shows potential for developing desirable food products in the future.

This increases the potential of using *A. filiculoides* and *A. pinnata* as a high calorie food to provide macronutrients to both livestock and human populations on a larger scale. Already lower TPC of *A. caroliniana* means that it is less limited in use than its congeners. As predicted, the TPC significantly decreased for all treatments from the control. However, given the relatively low TPC, cooking *A. caroliniana* in water may not be necessary and should be avoided to reduce the loss of nutrients associated with these cooking methods.

Boiling and pressure cooking reduced TPC the most and were not statistically different from one another. Both treatments reduced dry weight TPC by approximately 10‐fold. Natural fermentation was still effective in reducing dry weight TPC by about 2.5 times. Although protein content did increase on a dry weight bases from all postharvest treatments, there was protein yield loss from all postharvest treatments when looking at yield from 100 g of fresh *A. caroliniana*. In future experiments it is worth looking at differences in treatments and APD%, as this may make the protein loss due to such treatments negligible when compared by amount of digestible protein.

Expectedly, postharvest treatments either significantly decreased mineral content or had no effect. This suggests that if the goal of using *Azolla* is for providing micronutrients along with macronutrients, then it is not suggested that these postharvest treatments be performed as they all decreased mineral yield from *A. caroliniana*.

Future studies should focus more on nutritional value in other cooking methods that do not involve submersion in water to retain more nutrients, such as sautéing, steaming, or microwaving which may still reduce TPC while preserving other nutrients (Boari et al., 2013; Lozano‐Castellón et al., 2020; Ramírez‐Anaya et al., 2015). Future research should also focus on more in‐depth nutritional and genetic analyses to determine potentially useful cultivars and to develop large‐scale growth/harvest procedures. Additionally, improvements to the flavor of *Azolla* using different preparation and cooking methods are needed to further provide incentives for development and full domestication.

## 5 CONCLUSION

*Azolla caroliniana* holds excellent potential for use as a fast‐growing, short season crop that requires minimal inputs, upkeep, and processing. Our study highlights the nutritional value and moderate protein content of *A. caroliniana* and that low‐cost treatment methods easily and significantly reduce TPC. The TPC of raw *A. caroliniana* (4.26 g GAE kg^−1^ DW) is much lower than other species of *Azolla* (23.8–78.7 g GAE kg^−1^ DW), which means its use as an edible plant should not be limited by high TPC. *Azolla*'s moderate protein and high mineral yields make this species desirable for cultivation. The easy, fast‐growing nature of *Azolla* cultivation makes it an ideal resource during disasters, catastrophes, as well as regular use by smallholder farms and low‐income areas. Our study corroborates with others showing that members of the *Azolla* genus have high potential for economic, agricultural, nutritional, and resiliency benefits. *Azolla caroliniana* is a multipurpose valuable wild edible plant that shows great potential and needs further development.

## AUTHOR CONTRIBUTIONS

**Daniel Winstead:** Conceptualization (equal); data curation (lead); formal analysis (lead); investigation (equal); methodology (equal); project administration (equal); writing – original draft (lead); writing – review and editing (lead). **Francesco Di Gioia:** Data curation (supporting); methodology (equal); resources (equal); supervision (equal); writing – review and editing (equal). **Marjorie Jauregui:** Data curation (equal); methodology (equal); resources (equal); writing – review and editing (equal). **Michael Jacobson:** Conceptualization (equal); funding acquisition (lead); project administration (equal); resources (lead); supervision (supporting); writing – review and editing (equal).

## FUNDING INFORMATION

This research was funded by the Food Resilience in the Face of Catastrophic Global Events grant funded by the Open Philanthropy and FD contribution was supported by the USDA National Institute of Food and Agriculture and Hatch Appropriations under Project #PEN04723 and Accession #1020664.

## CONFLICT OF INTEREST STATEMENT

The authors declare no conflicts of interest.

## Data Availability

The data that support the findings of this study are openly available in figshare at https://doi.org/10.6084/m9.figshare.23296406.v1.

## References

[fsn33904-bib-0001] Abushita, A. A. , Daood, H. G. , & Biacs, P. A. (2000). Change in carotenoids and antioxidant vitamins in tomato as a function of varietal and technological factors. Journal of Agricultural and Food Chemistry, 48(6), 2075–2081. 10.1021/jf990715p 10888501

[fsn33904-bib-0002] Álvarez, R. , Araya, H. , Navarro‐Lisboa, R. , & de Dicastillo, C. L. (2016). Evaluation of polyphenol content and antioxidant capacity of fruits and vegetables using a modified enzymatic extraction. Food Technology and Biotechnology, 54(4), 462–467. 10.17113/ftb.54.04.16.4497 28115904 PMC5253986

[fsn33904-bib-0003] Boari, F. , Cefola, M. , Di Gioia, F. , Pace, B. , Serio, F. , & Cantore, V. (2013). Effect of cooking methods on antioxidant activity and nitrate content of selected wild Mediterranean plants. International Journal of Food Sciences and Nutrition, 64(7), 870–876. 10.3109/09637486.2013.799125 23701122

[fsn33904-bib-0004] Bravo, L. (1998). Polyphenols: Chemistry, dietary sources, metabolism, and nutritional significance. Nutrition Reviews, 56(11), 317–333. 9838798 10.1111/j.1753-4887.1998.tb01670.x

[fsn33904-bib-0005] Brouwer, P. , Schluepmann, H. , Nierop, G. J. , Elderson, J. , Bijl, P. K. , van der Meer, I. , de Visser, W. , Reichart, G. J. , Smeekens, S. , & van der Werf, A. (2018). Growing *Azolla* to produce sustainable protein feed: The effect of differing species and CO_2_ concentrations on biomass productivity and chemical composition. Journal of the Science of Food and Agriculture, 98, 4759–4768. 10.1002/jsfa.9016 29573358 PMC6099237

[fsn33904-bib-0006] Bunch, J. , & Hayden, W. J. (2020). Noteworthy collections: *Azolla filiculoides* new to Virginia. Castanea, 85(2), 229–231. 10.2179/0008-7475.85.2.229

[fsn33904-bib-0007] Carlozzi, P. , & Padovani, G. (2016). The aquatic fern *Azolla* as a natural plant‐factory for ammonia removal from fish‐breeding fresh wastewater. Environmental Science and Pollution Research, 23(9), 8749–8755. 10.1007/s11356-016-6120-8 26805923

[fsn33904-bib-0008] Cruz, Y. , Kijora, C. , Wedler, E. , Danier, J. , & Schulz, C. (2011). Fermentation properties and nutritional quality of selected aquatic macrophytes as alternative fish feed in rural areas of the Neotropics. Livestock Research for Rural Development, 23(11), 239–246.

[fsn33904-bib-0009] Debusk, W. F. , & Reddy, K. R. (1987). Growth and nutrient uptake potential of *Azolla caroliniana* Willd. And *Salvinia rotundifolia* Willd. as a function of temperature. Environmental and Experimental Botany, 27(2), 215–221. 10.1016/0098-8472(87)90072-4

[fsn33904-bib-0010] Dryden, G. W. , Song, M. , & McClain, C. (2006). Polyphenols and gastrointestinal diseases. Current Opinion in Gastroenterology, 22, 165–170. 10.1097/01.mog.0000208463.69266.8c 16462174 PMC4216723

[fsn33904-bib-0011] Everette, J. D. , Bryant, Q. M. , Green, A. M. , Abbey, Y. A. , Wangila, G. W. , & Walker, R. B. (2010). Thorough study of reactivity of various compound classes toward the Folin‐Ciocalteu reagent. Journal of Agricultural and Food Chemistry, 58(14), 8139–8144. 10.1021/jf1005935 20583841 PMC4075968

[fsn33904-bib-0012] Evrard, C. , & Van Hove, C. (2004). Taxonomy of the American *Azolla* species (Azollaceae): A critical review. Systematics and Geography of Plants, 74(2), 301–318.

[fsn33904-bib-0013] Fujihara, S. , Kasuga, A. , & Aoyagi, Y. (2001). Nitrogen‐to‐protein conversion factors for common vegetables in Japan. Journal of Food Science, 66(3), 412–415. 10.1111/j.1365-2621.2001.tb16119.x 18387100

[fsn33904-bib-0014] Giovanelli, G. , Zanoni, B. , Lavelli, V. , & Nani, R. (2002). Water sorption, drying and antioxidant properties of dried tomato products. Journal of Food Engineering, 52, 135–141.

[fsn33904-bib-0015] Hamdan, H. Z. , & Houri, A. F. (2022). CO_2_ sequestration by propagation of the fast‐growing *Azolla* spp. Environmental Science and Pollution Research, 29(12), 16912–16924. 10.1007/s11356-021-16986-6 34657254 PMC8520330

[fsn33904-bib-0016] Haminiuk, C. W. I. , Plata‐Oviedo, M. S. V. , de Mattos, G. , Carpes, S. T. , & Branco, I. G. (2014). Extraction and quantification of phenolic acids and flavonols from *Eugenia pyriformis* using different solvents. Journal of Food Science and Technology, 51(10), 2862–2866. 10.1007/s13197-012-0759-z 25328239 PMC4190214

[fsn33904-bib-0017] Hassan, A. M. , Mohamed, H. E. , & Mostafa, E. M. (2020). Nutrient starvation enhances the phenolic compounds and antioxidant activity in *Azolla caroliniana* plant. Egyptian Journal of Botany, 60(1), 239–247. 10.21608/ejbo.2019.15970.1351

[fsn33904-bib-0018] Hsu, H. W. , Vavak, D. L. , Satterlee, L. D. , & Miller, G. A. (1977). A multienzyme technique for estimating protein digestibility. Journal of Food Science, 42(5), 1269–1273. 10.1111/j.1365-2621.1977.tb14476.x

[fsn33904-bib-0019] Huang, C. L. , & Schulte, E. E. (1985). Digestion of plant tissue for analysis by ICP emission spectroscopy. Communications in Soil Science and Plant Analysis, 16(9), 943–958. 10.1080/00103628509367657

[fsn33904-bib-0020] Hundare, S. K. , Pathan, D. I. , & Ranadive, A. B. (2018). Use of fermented Azolla in diet of tilapia fry (*Oreochromis niloticus*). International Journal of Bio‐Resource and Stress Management, 9(6), 702–706. 10.23910/IJBSM/2018.9.6.1925

[fsn33904-bib-0021] Katayama, N. , Yamashita, M. , Kishida, Y. , Liu, C. C. , Watanabe, I. , & Wada, H. (2008). *Azolla* as a component of the space diet during habitation on Mars. Acta Astronautica, 63(7–10), 1093–1099. 10.1016/j.actaastro.2008.01.023

[fsn33904-bib-0022] Kaur, H. , Dhawan, A. , & Ansal, M. D. (2015). Harvesting management for protein rich biomass production from Azolla (*Azolla caroliniana*). Indian Journal of Animal Nutrition, 32(3), 320–324.

[fsn33904-bib-0023] Liu, A. G. , Ford, N. A. , Hu, F. B. , Zelman, K. M. , Mozaffarian, D. , & Kris‐Etherton, P. M. (2017). A healthy approach to dietary fats: Understanding the science and taking action to reduce consumer confusion. Nutrition Journal, 16, 53. 10.1186/s12937-017-0271-4 28854932 PMC5577766

[fsn33904-bib-0024] Lozano‐Castellón, J. , Vallverdú‐Queralt, A. , de Alvarenga, J. F. R. , Illán, M. , Torrado‐Prat, X. , & Lamuela‐Raventós, R. M. (2020). Domestic sautéing with EVOO: Change in the phenolic profile. Antioxidants, 9(1), 77. 10.3390/antiox9010077 31963124 PMC7022658

[fsn33904-bib-0025] Marles, R. J. (2017). Mineral nutrient composition of vegetables, fruits and grains: The context of reports of apparent historical declines. Journal of Food Composition and Analysis, 56, 93–103. 10.1016/j.jfca.2016.11.012

[fsn33904-bib-0026] McLachlan, M. , & Landman, A. P. (2013). Nutrition‐sensitive agriculture—A South African perspective. Food Security, 5(6), 857–871. 10.1007/s12571-013-0309-1

[fsn33904-bib-0027] Mennen, L. I. , Walker, R. , Bennetau‐Pelissero, C. , & Scalbert, A. (2005). Risks and safety of polyphenol consumption. The American Journal of Clinical Nutrition, 81(Suppl), 326S–329S. 15640498 10.1093/ajcn/81.1.326S

[fsn33904-bib-0028] Miranda, A. F. , Biswas, B. , Ramkumar, N. , Singh, R. , Kumar, J. , James, A. , Roddick, F. , Lal, B. , Subudhi, S. , Bhaskar, T. , & Mouradov, A. (2016). Aquatic plant Azolla as the universal feedstock for biofuel production. Biotechnology for Biofuels, 9, 221. 10.1186/s13068-016-0628-5 27777623 PMC5069886

[fsn33904-bib-0029] Muradov, N. , Taha, M. , Miranda, A. F. , Kadali, K. , Gujar, A. , Rochfort, S. , Stevenson, T. , Ball, A. S. , & Mouradov, A. (2014). Dual application of duckweed and azolla plants for wastewater treatment and renewable fuels and petrochemicals production. Biotechnology for Biofuels, 7, 30. 10.1186/1754-6834-7-30 24576349 PMC3944989

[fsn33904-bib-0030] Nuraini, N. , Mirzah, M. , Nur, Y. S. , & Harnentis, H. (2022). Improving *Azolla microphylla* through fermentation with lignocellulolytic fungi and its application in broiler feed. Advances in Animal and Veterinary Sciences, 10(5), 1090–1100. 10.17582/JOURNAL.AAVS/2022/10.5.1090.1100

[fsn33904-bib-0031] Polat, S. , Guclu, G. , Kelebek, H. , Keskin, M. , & Selli, S. (2022). Comparative elucidation of colour, volatile and phenolic profiles of black carrot (*Daucus carota* L.) pomace and powders prepared by five different drying methods. Food Chemistry, 369, 130941. 10.1016/j.foodchem.2021.130941 34479009

[fsn33904-bib-0032] Poudel, P. , Di Gioia, F. , Lambert, J. D. , & Connolly, E. L. (2023). Zinc biofortification through seed nutri‐priming using alternative zinc sources and concentration levels in pea and sunflower microgreens. Frontiers in Plant Science, 14, 1177844. 10.3389/fpls.2023.1177844 37139105 PMC10150129

[fsn33904-bib-0033] Pouil, S. , Samsudin, R. , Slembrouck, J. , Sihabuddin, A. , Sundari, G. , Khazaidan, K. , Kristanto, A. H. , Pantjara, B. , & Caruso, D. (2020). Effects of shading, fertilization and snail grazing on the productivity of the water fern *Azolla filiculoides* for tropical freshwater aquaculture. Aquatic Botany, 160, 103150. 10.1016/j.aquabot.2019.103150

[fsn33904-bib-0034] Price, K. R. , Bacon, J. R. , & Rhodes, M. J. C. (1997). Effect of storage and domestic processing on the content and composition of flavonol glucosides in onion (*Allium cepa*). Journal of Agricultural and Food Chemistry, 45(3), 938–942. 10.1021/jf9605916

[fsn33904-bib-0035] Ramírez‐Anaya, J. D. P. , Samaniego‐Sánchez, C. , Castañeda‐Saucedo, M. C. , Villalón‐Mir, M. , & De La Serrana, H. L. G. (2015). Phenols and the antioxidant capacity of Mediterranean vegetables prepared with extra virgin olive oil using different domestic cooking techniques. Food Chemistry, 188, 430–438. 10.1016/j.foodchem.2015.04.124 26041214

[fsn33904-bib-0036] Rickman, J. C. , Bruhn, C. M. , & Barrett, D. M. (2007). Nutritional comparison of fresh, frozen, and canned fruits and vegetables II. Vitamin a and carotenoids, vitamin E, minerals and fiber. Journal of the Science of Food and Agriculture, 87, 1185–1196. 10.1002/jsfa.2824

[fsn33904-bib-0037] Ritchie, H. , Roser, M. , & Rosado, P. (2022). Crop yields. Our World in Data https://ourworldindata.org/crop‐yields

[fsn33904-bib-0038] Sahlin, E. , Savage, G. P. , & Lister, C. E. (2004). Investigation of the antioxidant properties of tomatoes after processing. Journal of Food Composition and Analysis, 17(5), 635–647. 10.1016/j.jfca.2003.10.003

[fsn33904-bib-0039] Samtiya, M. , Aluko, R. E. , & Dhewa, T. (2020). Plant food anti‐nutritional factors and their reduction strategies: An overview. Production, Processing and Nutrition, 2, 6.

[fsn33904-bib-0040] Samtiya, M. , Aluko, R. E. , Puniya, A. K. , & Dhewa, T. (2021). Enhancing micronutrients bioavailability through fermentation of plant‐based foods: A concise review. Fermentation, 7, 63. 10.3390/fermentation7020063

[fsn33904-bib-0041] Sjödin, E. (2012). The Azolla Cooking and Cultivation Project . Retrieved from http://www.eriksjodin.net

[fsn33904-bib-0042] Small, E. (2013). North American Cornucopia. CRC Press.

[fsn33904-bib-0043] Stewart, A. J. , Bozonnet, S. , Mullen, W. , Jenkins, G. I. , Lean, M. E. J. , & Crozier, A. (2000). Occurrence of flavonols in tomatoes and tomato‐based products. Journal of Agricultural and Food Chemistry, 48(7), 2663–2669. 10.1021/jf000070p 10898604

[fsn33904-bib-0044] Turkmen, N. , Sari, F. , & Velioglu, Y. S. (2005). The effect of cooking methods on total phenolics and antioxidant activity of selected green vegetables. Food Chemistry, 93(4), 713–718. 10.1016/j.foodchem.2004.12.038

[fsn33904-bib-0045] Ur‐Rehman, Z. , Islam, M. , & Shah, W. H. (2003). Effect of microwave and conventional cooking on insoluble dietary fibre components of vegetables. Food Chemistry, 80, 237–240.

[fsn33904-bib-0046] van Het Hof, K. H. , West, C. E. , Weststrate, J. A. , & Hautvast, J. G. (2000). Dietary factors that affect the bioavailability of carotenoids. Journal of Nutrition, 130(3), 503–506. 10702576 10.1093/jn/130.3.503

[fsn33904-bib-0047] Vecchia, L. , Di Gioia, F. , Ferrante, A. , Hong, J. C. , White, C. , & Rosskopf, E. N. (2020). Integrating cover crops as a source of carbon for anaerobic soil disinfestation. Agronomy, 10, 1614. 10.3390/agronomy10101614

[fsn33904-bib-0048] Vincenzini, M. , Margheri, M. C. , & Sili, C. (1985). Outdoor mass culture of *Azolla* spp.: Yields and efficiencies of nitrogen fixation. Plant and Soil, 86(1), 57–67.

[fsn33904-bib-0049] Wagner, G. M. (1997). *Azolla*: A review of its biology and utilization. Botanical Review, 63(1), 1–26. 10.1007/BF02857915

[fsn33904-bib-0050] Waterman, P. G. , & Mole, S. (1994). Analysis of phenolic plant metabolites. Blackwell Scientific Publications.

[fsn33904-bib-0051] Winstead, D. J. , Jacobson, M. G. , & Di Gioia, F. (2023). Valorizing staple native American food plants as a food resilience resource. Frontiers in Sustainable Food Systems, 7, 1117805. 10.3389/fsufs.2023.1117805

[fsn33904-bib-0052] Yao, Y. , Zhang, M. , Tian, Y. , Zhao, M. , Zeng, K. , Zhang, B. , Zhao, M. , & Yin, B. (2018). Azolla biofertilizer for improving low nitrogen use efficiency in an intensive rice cropping system. Field Crops Research, 216, 158–164. 10.1016/j.fcr.2017.11.020

[fsn33904-bib-0053] Zhang, D. , & Hamauzu, Y. (2004). Phenolics, ascorbic acid, carotenoids and antioxidant activity of broccoli and their changes during conventional and microwave cooking. Food Chemistry, 88(4), 503–509. 10.1016/j.foodchem.2004.01.065

